# Selective targeted delivery of the TNF-alpha receptor p75 and uteroglobin to the vasculature of inflamed tissues: a preliminary report

**DOI:** 10.1186/1472-6750-11-104

**Published:** 2011-11-10

**Authors:** Elisa Ventura, Enrica Balza, Laura Borsi, Giorgia Tutolo, Barbara Carnemolla, Patrizia Castellani, Luciano Zardi

**Affiliations:** 1 Laboratory of Therapeutic Recombinant Proteins, Centro Biotecnologie Avanzate, Largo Rosanna Benzi, 1016132 Genoa, Italy; 2 Laboratory of Oncology, G. Gaslini Institue, 16147 Genoa, Italy; 3 Laboratory of Cell Biology and Immunology, National Institute for Research on Cancer, Largo Rosanna Benzi, 1016132 Genoa, Italy

## Abstract

**Background:**

Ligand-targeted approaches have proven successful in improving the therapeutic index of a number of drugs. We hypothesized that the specific targeting of TNF-alpha antagonists to inflamed tissues could increase drug efficacy and reduce side effects.

**Results:**

Using uteroglobin (UG), a potent anti-inflammatory protein, as a scaffold, we prepared a bispecific tetravalent molecule consisting of the extracellular ligand-binding portion of the human TNF-alpha receptor P75 (TNFRII) and the scFv L19. L19 binds to the ED-B containing fibronectin isoform (B-FN), which is expressed only during angiogenesis processes and during tissue remodeling. B-FN has also been demonstrated in the pannus in rheumatoid arthritis. L19-UG-TNFRII is a stable, soluble homodimeric protein that maintains the activities of both moieties: the immuno-reactivity of L19 and the capability of TNFRII to inhibit TNF-alpha. *In vivo *bio-distribution studies demonstrated that the molecule selectively accumulated on B-FN containing tissues, showing a very fast clearance from the blood but a very long residence time on B-FN containing tissues. Despite the very fast clearance from the blood, this fusion protein was able to significantly improve the severe symptomatology of arthritis in collagen antibody-induced arthritis (CAIA) mouse model.

**Conclusions:**

The recombinant protein described here, able to selectively deliver the TNF-alpha antagonist TNFRII to inflamed tissues, could yield important contributions for the therapy of degenerative inflammatory diseases.

## Background

One of the key inflammatory mediators in chronic inflammatory processes is the cytokine tumour necrosis factor-alpha (TNF-alpha). In fact, it has been demonstrated that TNF-alpha plays a role in various inflammatory diseases such as rheumatoid arthritis (RA), ankylosing spondylitis, psoriasis, Crohn's disease, psoriatic arthritis, and juvenile idiopathic arthritis [1]. This observation led to the generation of TNF-alpha antagonists such as Infliximab (trade name REMICADE) a mouse-human chimeric monoclonal, etanercept (trade name ENBREL) a TNF-alpha receptor p75-IgG fusion protein and adalimumab (trade name HUMIRA) a fully human IgG1 monoclonal antibody. Each of these three antagonists binds to TNF-alpha, thereby preventing it from activating its receptor. The use of these TNF-alpha inhibitors has led to dramatic clinical improvements in the treatment of the above-mentioned diseases [[1,2] and references therein]].

The major therapeutic goal when administering TNF-alpha inhibitors is to neutralize the surplus of TNF-alpha both from the blood circulation and from inflamed tissues. In RA higher than normal TNF levels have been detected in both serum and arthritic joints. In Crohn's disease TNF concentration is abnormally high in serum as well as in the gut mucosa. In psoriasis elevated TNF levels have been measured in serum and in the epidermis of psoriatic plaques [[1,2] and references therein]. While TNF-alpha present in the bloodstream can be annulled without particular problems, its neutralization in inflamed tissues requires the effective penetration and an optimal concentration of the drug in the diseased tissues. To achieve this high doses of the TNF-alpha inhibitor would be needed, but these would be accompanied by severe side effects [3]. A possible solution to this problem is the generation of small molecules with enhanced penetration into tissues. Neve et al. first investigated this possibility in 1994 by substituting the Fc portion of IgG present in Etanercept with a smaller linker[4]. However the fast blood clearance of a small molecule is very likely to reduce its therapeutic performance.

Another attractive possibility is the targeted delivery of the TNF-alpha antagonist directly to inflamed tissues by fusing the agents with a ligand that can to selectively transfer the drug to a specific target. A long residence time of the drug in the target should overcome the problems caused by the fast blood clearance. The selection of the target is crucial for this kind of system. A recent paper describes an elegant approach of targeting arthritic cartilage using an scFv specific for ROS (reactive oxygen species) modified collagen II. In fact there is an excessive level of ROS in RA. Using a fusion protein constituted by the scFv to ROS-modified collagen II and TNFRII they report that it significantly reduced inflammation in arthritic mice compared to the TNF-RII-Fc alone [5].

As a target we chose B-FN, a fibronectin (FN) isoform containing the extra-domain B (ED-B), is undetectable in the tissues of healthy adults. By contrast, it is significantly up regulated in foetal and tumour tissues and all angiogenesis-associated diseases including inflammatory degenerative pathologies [6-8]. In RA B-FN has been demonstrated in the pannus [9]. The pannus is a highly vascularised inflammatory granulation tissue that spreads from the synovial membrane and invades the joints. Furthermore, B-FN has been associated with the invasive phenotype described in RA. Previous studies have demonstrated that a fusion protein composed of IL10 and the anti-ED-B scFv L19 selectively accumulates in the RA-affected joints in a mouse experimental model [10,11]. More recently, it has been demonstrated that a human recombinant antibody to B-FN, conjugated to a near infrared dye, selectively accumulates in the inflamed joints of the collagen antibody-induced arthritis (CAIA) mouse model, which enables fluorescence imaging of RA affected joints *in vivo *[12].

These observations have prompted our investigation into approaches that allow the selective delivery of TNF-alpha antagonists to RA-affected joints. We generated a tetravalent dual specific fusion protein composed of the active moiety of Etanercept, TNFRII, and the scFv L19, a human scFv to ED-B which is currently used extensively in preclinical studies as well clinical trials to target B-FN in different kind of cancers [13,14]. To construct the L19-TNFRII fusion protein we used uteroglobin (UG), a small (15.8 kDa), globular, non-glycosilated and homodimeric protein [15], as a scaffold. We have previously reported that the use of UG as a scaffold improves properties of solubility and stability of recombinant fusion proteins. In addition, since UG generates a covalently linked homodimer, we demonstrated that it is possible to generate dual specific tetravalent molecules using a single cDNA construct [15,16]. Using UG as a linker, different kinds of fusion proteins have been generated that, compared to similar fusion proteins without UG, possess enhanced properties of solubility and stability, factors that expedite their storage and clinical uses. Moreover, UG contains a central hydrophobic cavity able to accommodate hydrophobic molecules such as progesterone, retinol and prostaglandin D2. Theoretically this cavity could be loaded with different types of therapeutic substances and delivered to the target. Furthermore, the use of UG as a scaffold is not only a flexible and robust procedure for the generation of dual specific tetravalent fusion proteins, but, given the potent anti-inflammatory properties of UG, it is the linker of choice for the generation of fusion proteins for therapeutic uses in inflammatory diseases [13]. In fact, UG has been demonstrated capable of inhibiting the secretory phosholipase A2, a key enzyme in the production of diverse mediators of inflammation that plays a crucial role in inflammatory diseases like RA [15,17]. A synthetic peptides called Antiflammin, derived from UG, is one of the most potent anti-inflammatory agents and has been used to generate powerful anti-inflammatory molecules [18-21]. Furthermore, UG can modulate tissue transglutaminase. In fact, in an experimental model of crescentic glomerulonepritis, UG was able to attenuate renal inflammation by modulating the expression of tissue transglutaminase [22,23].

This paper reports on the production and characterization of the fusion protein L19-UG-TNFRII and its ability to selectively accumulate *in vivo *in B-FN-containing tissues and to significantly improve the objective signs of arthritis in the in CAIA mouse model.

## Results

### Expression, Purification And Characterization of L19-UG-TNFRII Fusion Protein

**Figure 1:**
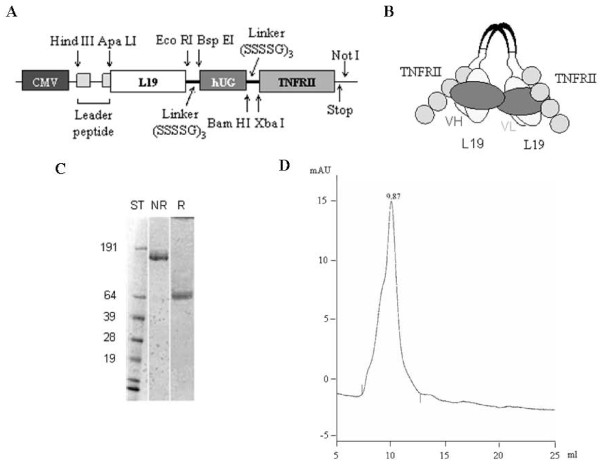
**L19-hUG-TNFRII characterization**. A) Scheme of the cDNA of L19-UG-TNFRII including the leader peptide; CMV cytomegalovirus promoter. B) Schematic representation of homodimeric L19-UG-TNFRII. C) SDS-PAGE analysis of purified L19-UG-TNFRII under reducing (R) and non-reducing (NR) conditions; on the left the molecular masses of the standards in kDa. D) Size exclusion chromatography profile (Superdex 200) of purified L19-UG-TNFRII; mAU, milli-absorbance units.

We generated a dual specificity tetravalent molecule, L19-UG-TNFRII, using human UG to link the extracellular ligand-binding portion of the human TNF-alpha receptor 75 (p75) (TNFRII) with the scFv L19 specific for the ED-B domain of FN (Figure 1B). We appended the cDNA of the scFv L19 and the cDNA of TNFRII at the 5' and 3'ends of the UG cDNA, respectively (Figure 1A). The construct was inserted into a mammalian expression vector and used to transfect CHO cells. The fusion protein was purified from the conditioned media of the transfected cells by affinity chromatography as described in "Methods".

The purified fusion protein was then analyzed in SDS-PAGE (Figure 1C) in which it migrated as a monomer of the expected size of about 60 KDa in reducing conditions, while it migrated as a homodimer with an apparent molecular mass of about 120 kDa under non-reducing conditions. The size exclusion chromatography profile (Superdex 200) showed a single peak with a retention volume corresponding to the apparent molecular mass of the homodimer and the absence of any aggregate (Figure 1D).

**Figure 2:**
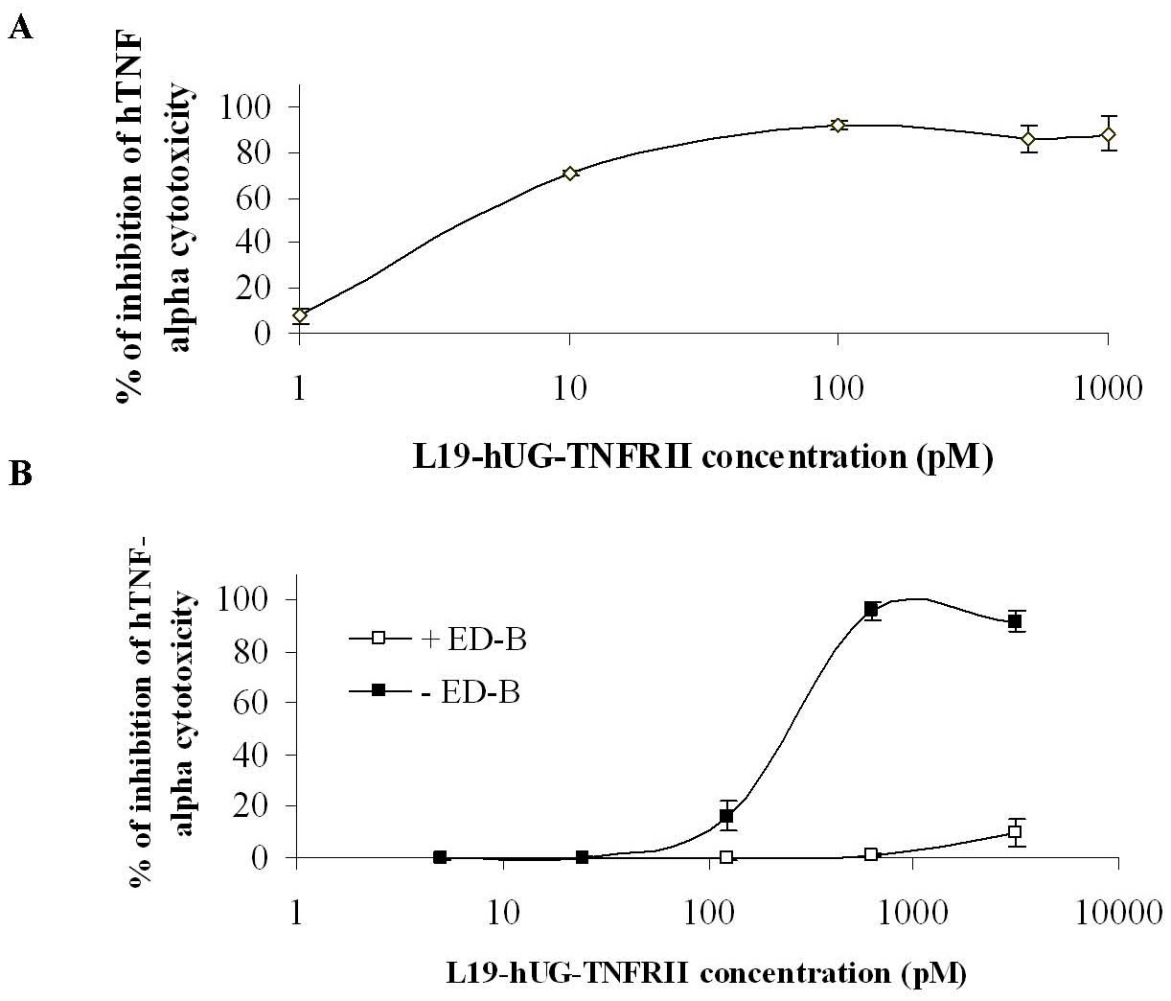
***In vitro *L19-hUG-TNFRII biological activity**. A) Inhibitory activity of TNF-alpha cytotoxicity by different concentrations of L19-UG-TNFRII using LM mouse fibroblasts treated with 2 pM TNF-alpha. B) L19-UG-TNFRII bound to ED-B is able to inhibit TNF-alpha cytotoxicity (*in situ *inhibition). The cytotoxic activity of 25 pM TNF-alpha was evaluated on LM cells using plates pre-coated with the recombinant FN fragment 7.ED-B.8.9 and pre-incubated with different concentrations of L19-UG-TNFRII (5-3125 pM). To assess the role of the L19 component the experiment was also carried out mixing the L19-UG-TNFRII with a large excess of ED-B to inhibit of the scFv L19 moiety. After washing out the unbound fusion protein, TNF-alpha was inhibited only by L19-UG-TNFRII bound to the recombinant FN fragment with which the plates were coated. The means ± S.D. are reported.

The purified fusion protein was tested for immunoreactivity of the scFv moiety by immunohistochemistry and ELISA and the same immunoreactivity of the original scFv was found (data not shown). L19-UG-TNFRII was also evaluated for the biological activity of the TNFRII components by measuring its ability to inhibit TNF-alpha using a cytotoxic assay on LM mouse fibroblasts. As shown in Figure 2A the TNF-alpha neutralizing capabilities of TNFRII were preserved in the fusion protein, thereby demonstrating that the scFv L19 and TNFRII within the fusion protein do not interfere with each other.

To demonstrate that L19-UG-TNFRII inhibits TNF-alpha activity even when it is bound to ED-B, we mimicked the targeted delivery of L19-UG-TNFRII to tissues containing B-FN, running citotoxicity experiments on LM cells seeded on plates pre-coated with the FN recombinant fragment 7.ED-B.8.9. After incubating the cells with L19-UG-TNFRII and washing out the excess, TNF-alpha was added. Under this condition only the L19-UG-TNFRII bound to the B-FN recombinant fragment is present on the plates. The results, shown in Figure 2B, demonstrated that even when L19-UG-TNFRII is bound to ED-B, the TNFRII moiety is still able to neutralize TNF-alpha cytotoxicity. On the contrary when L19-UG-TNFRII was premixed with a large excess of recombinant ED-B to neutralize the L19 moiety of the fusion protein, no inhibition of the cytotoxicity activity of TNF-alpha was observed (Figure 2B).

### L19-UG-TNFRII selectively accumulates in B-FN containing tissues

To establish whether L19-UG-TNFRII selectively accumulated on B-FN containing tissues, we ran bio-distribution experiments using ^125^I radio-iodinated recombinant protein. As a model of B-FN containing tissue we use the teratocarcinoma mouse model F9, since it has been extensively evaluated for targeting of the scFv L19 and it is possible to obtain quantitative results.

The radio-iodinated fusion protein was *iv *injected in F9-tumour bearing mice; groups of 3 animals were sacrificed at 4, 24 and 48 hours after injection.

**Figure 3:**
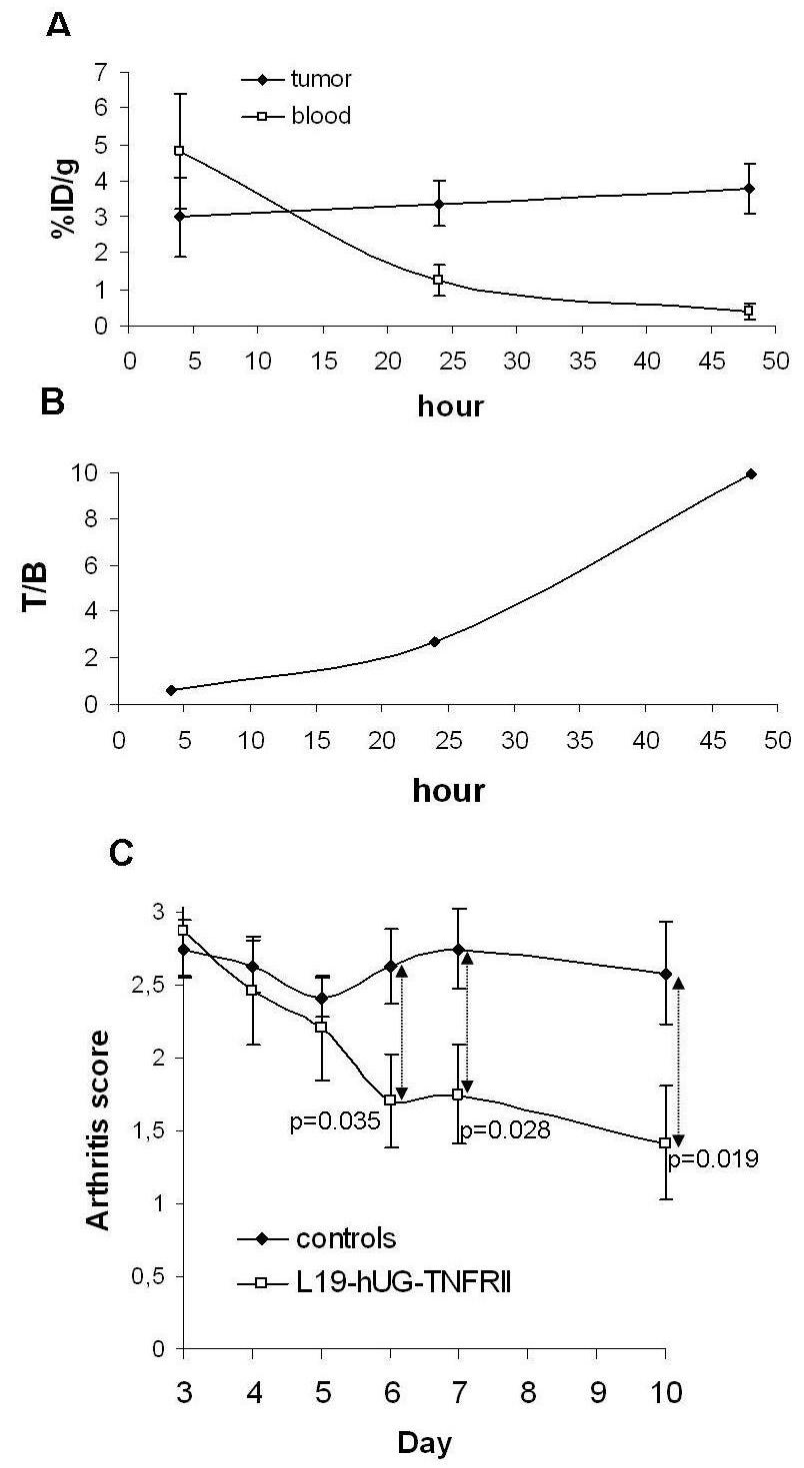
***In vivo *L19-hUG-TNFRII bio-distribution and therapy properties**. A-B) Bio-distribution of the radio-iodinated L19-UG-TNFRII in F9 teratocarcinoma-bearing mice. A) The %ID/g in the tumour and in the blood at the indicated times after i.v. injection of the radio-iodinated L19-UG-TNFRII are shown (mean ± S.D.). B) The tumour to blood ratio of the %ID/g at different times after injection of the radio-iodinated protein are plotted. C) *In vivo *therapy experiments with L19-UG-TNFRII in CAIA animal models. The median arthritis scores determined in treated and control mice at day 3 to 10 from the injection of anti-collagen antibodies are indicated. The p values determined using the non parametric Mann-Whitney U test are indicated. Bars indicate the standard error of the mean.

^125^I-L19-UG-TNFRII showed a selective, high and very stable accumulation in tumours. 48 hours after iv injection the %ID/g of the radio-iodinated protein in tumour was 4, while it was 0.1 in the blood and lower then 0.4 in any other organs (data not shown). Figure 3A shows that while the %ID/g in the tumour increased with the increase of time from the injection, the %ID/g in blood quickly decreased. In fact, blood clearance of L19-UG-TNFRII was mediated mainly by way of the kidneys, as determined by evaluating urine samples, and showed a biphasic curve with an alpha and a beta phase. Despite a molecular size of nearly 140 KDa, the alpha and beta phase half-life of L19-UG-TNFRII were of about 1 hour and 20 hours respectively.

### L19-UG-TNFRII reduces the signs of arthritis in the in Collagen Antibody-Induced Arthritis (CAIA) mouse model

Arthritis was induced in mice using the Collagen Antibody-Induced Arthritis (CAIA) with single intravenous injections of 2 mg of a cocktail of five anti-type II collagen monoclonal antibodies. We observed evident signs of severe arthritis in all animals on day 3, with a median arthritis score of about 3. Figure 3B shows the therapeutic results obtained by treating the animals with 50 μg of ip administered L19-hUG-TNFRII given daily from day 3 to day 7. Treatment with L19-UG-TNFRII was effective in improving the severity of the arthritis. We observed improvement in the involved paws in all treated animals, with redness and swelling of the inflamed paws being markedly reduced. On the contrary, the severity of the disease was persistent in untreated mice.

Statistical comparison using the Mann-Whitney test revealed highly significant differences in the scores of treated and untreated animals showing a p < 0.03 on day 7 and p < 0.02 on day 10 (Figure 3B).

## Discussion

The ECM is essential for the normal organization and function of all tissues and provides specific environmental signals to control cell morphology, migration, differentiation, and proliferation. Thus, healthy normal tissues that, for physiological or pathological causes, undergo remodeling are subject to profound modifications in ECM components in response to the mutated physiological requirements. Indeed, at the onset and during progression of major diseases including cancer, atherosclerosis, and rheumatoid arthritis, the ECM composition is altered by two main processes: proteolytic degradation of the normal tissue's pre-existing ECM components and neo-synthesis of new ECM components. The interplay between these two processes results in the generation of a provisional ECM whose components may differ, qualitatively and quantitatively, from their healthy counterparts. This provisional ECM provides a permissive environment for disease progression, of which angiogenesis is a pivotal step. Thus, some disease-associated-ECM-proteins provide an attractive target for the selective delivery of drugs, and various proteins have been investigated precisely for this purpose [24].

B-FN has emerged as a prototypic component of the provisional ECM that can be used for the selective delivery of drugs to diseased tissues. The extra domain B is a complete type III homology repeat of 91 amino acids, in which exon usage or skipping leads to inclusion or exclusion of these type III repeat [6]. It is noteworthy that the pre-mRNA alternative splicing of a number of ECM components is cell cycle dependent and modulated by cytokines and pH [25-28].

The ED-B sequence is highly conserved in different species, having 100% homology in all mammalians thus far studied and 96% homology with a similar domain in chicken. The use of a murine mAb, BC-1 [7], selectively specific for B-FN, allowed immunohistochemistry studies on different tissues. Results demonstrated that B-FN is undetectable in the tissues of healthy adults with very rare exceptions, such as the recurrent physiological processes of tissue remodeling and angiogenesis occurring in the female reproductive system. B-FN is, in contrast, noticeably up regulated in foetal and tumour tissues as well as all angiogenesis-associated diseases, including degenerative inflammatory pathologies. Since B-FN accumulates around new-forming vessels, it is also an excellent marker of angiogenesis [8].

The demonstration that the mAb BC1 could be used to selectively target tumours *in vivo *prompted the generation of high-affinity human recombinant antibodies specific for the ED-B for diagnostic and therapeutic purposes. In particular, the scFv L19 [29] has been investigated mainly for cancer therapy, leading to preclinical and clinical investigations of several antibody-cytokine fusion proteins.

Here we report on the preparation of a fusion protein composed of the scFv L19 and TNFRII. To generate a protein that was bivalent for both L19 and TNFRII we adopted the recently described approach using UG as a central core of the fusion protein [16]. Human UG is a 15.8-kDa homodimeric globular non-glycosylated and homodimeric secreted protein. The two subunits are joined in an anti-parallel fashion by disulfide bridges established between two highly conserved cysteine residues in the amino- and carboxyl-terminal positions. The exact functions of UG are not yet clear, but the protein has been reported to have anti-inflammatory properties in view of its ability to inhibit the soluble phospholipase A2 and tissue transglutaminase [15]. This approach allowed the generation of a dual-specificity tetravalent molecule using a single cDNA construct. Furthermore, the use of UG in fusion proteins ameliorates properties of solubility and stability [16].

To ascertain whether L19-UG-TNFRII targeted B-FN *in vivo *we used the murine teratocarcinoma experimental model F9, which has been extensively studied to formally demonstrate the *in vivo *ability of the scFv to selectively accumulate on tissues containing B-FN. Results demonstrate that L19-UG-TNFRII selectively accumulates in the neoplasia and not in any of the non-neoplastic tissues. Furthermore, we observed a very rapid clearance from the blood, but a long residence time in the B-FN containing tissue. The fast clearance may reduce the toxicity of the molecules in the non-targeted organs, while the long residence time in B-FN containing tissues is useful for the therapeutic functions of the molecule.

We have chosen a tumour model to demonstrate the selective accumulation of L19-UG-TNFRII fusion protein on B-FN *in vivo*, to obtain quantitative results that cannot be achieved using a model of RA in mice. In fact, all the tumour mass contains B-FN it is easily and completely removable, weighable and countable in a gamma-counter. On the contrary, it is practically impossible to selectively remove the diseased tissues in RA models and have quantitative results such as the %ID/g.

We then carried out a study on the therapeutic activity of the L19-UG-TNFRII using the CAIA mouse model. Although the clearance was very fast compared to the half-life of about 100 hrs of etanercept, we observed very significant results in the reduction of rheumatoid arthritis symptoms. This could be due to the selective accumulation of TNFRII in the hypervascularized arthritic joints containing high quantities of B-FN.

We realize that much more extensive preclinical studies are needed, in particular comparative studies between targeted and non-targeted TNFRII, as well as combination studies using non-targeted TNFRII to neutralize blood TNF-alpha and L19-UG-TNFRII to neutralize tissue TNF-alpha; this combination should allow reduction of the total amount of administered TNFRII, thereby leading to a reduction of side effects. The anti-inflammatory potential of the UG component in the fusion protein must also be evaluated, and this should be possible using the fusion protein L19-UG that we have recently described [16]. The therapeutic efficacy of the targeted delivery of TNF-alpha antagonists to the inflamed tissues may thus provide a fertile future research area and yield important contributions for the therapy of degenerative inflammatory diseases.

## Conclusion

Using UG as a scaffold and the FN isoform containing the ED-B as a target, we have generated a fusion protein (L19-UG-TNFRII) able to selectively deliver a TNF-alpha inhibitor to inflamed tissues and we demonstrated that this fusion protein was able to significantly improve the severe symptomatology of arthritis in collagen antibody-induced arthritis (CAIA) mouse model.

Furthermore using this platform, an antibody specific for a component of the ECM of inflamed tissues, UG as a scaffold and an anti-inflammatory molecule as effector, it is possible to generate various fusion proteins that could yield important contributions for the therapy of degenerative inflammatory diseases.

## Methods

### Preparation of cDNA construct, expression, purification and characterization of L19-hUG-TNFRII

The cDNA encoding for L19 fused to human UG and to the extracellular ligand-binding portion of the human 75 KDa (p75) tumour necrosis factor receptor (TNFRII), optimized for expression in Chinese hamster ovary (CHO) cells and cloned into pcDNA3.1 mammalian expression vector (Invitrogen, Carlsbad, CA, USA), was provided by Genscript Corporation (Piscataway, NJ, USA).

The cDNA construct was used to transfect mammalian CHO cells (American Tissue Type Culture Collection, Manassas, VA) with the use of Lipofectamine 2000 CD Reagent (Invitrogen) according to the manufacturer's instructions. Cells were grown in RPMI 1640 (Sigma-Aldrich, St Louis, MO, USA) supplemented with 10% foetal bovine serum (Sigma) and 4 mM L-glutamine (Invitrogen).

L19-UG-TNFRII was purified from the spent media of the transfected cells by immunoaffinity chromatography on the recombinant extra-domain B (ED-B) fragment of FN [30] conjugated to Sepharose 4B (GE Healthcare, Uppsala, Sweden).

The purified protein was analysed by sodium dodecyl sulfate-plyacrylamide gel electrophoresis (SDS-PAGE; 4%-12%) under reducing and non-reducing conditions and in native conditions by fast-protein liquid chromatography (FPLC) on a S200 chromatography column.

The biological activity *in vitro *of the recombinant protein was assessed by determining the inhibition of human TNF-alpha cytotoxicity on mouse L-M fibroblasts (ATCC) as previously described [16].

### Radio-iodination and bio-distribution experiments of L19-UG-TNFRII

L19-UG-TNFRII was radio-iodinated as previously described [24] using the Iodogen method (Pierce, Rockford, IL, USA). 129/SvHsd mice (Harlan Italy, Udine, Italy) with subcutaneously implanted 3 × 10^6 ^F9 murine syngenic teratocarcinoma cells, were injected intravenously with roughly 5 μg (6,4 μCi, 0,237 MBq) of protein in 100 μl saline solution. Mice were sacrificed at 4, 24 and 48 hr after injection. The organs and the tumour were weighed and the radioactivity was counted in a gamma counter. Targeting results are expressed as a percent of the injected dose per gram of tissue (%ID/g). Housing, treatment and sacrifice of animals complied with national legislative provisions (Italian law no. 116 of 27 January, 1992).

### In vivo experiments in Collagen Antibody-Induced Arthritis (CAIA) models

Adult male DBA/1J mice were purchased from Harlan (Oxon UK). Arthritis was induced by a single intravenous injection of 2 mg of Arthrogen-CIA Arthritogenic Monoclonal Antibody Cocktail (Chondrex, Redmond, WA, USA) on day 0. On day 3, animals were randomly divided into two groups of six animals each; the first group received daily intraperitoneal injections of 50 μg of L19-UG-TNFRII dissolved in 20 mM sodium-phosphate buffer pH 7.6 containing 0.15 M NaCl. Animals were treated from day 3 to day 7. The second set of animals was used as untreated control group.

Animals were weighed daily and monitored for the onset and progression of disease from day 0 to day 10. Paws were assigned a clinical score between 0 and 4 according to the index: 0 = no arthritis; 1 = mild involvement in a single area (single interphalangeal joint, mid-foot or wrist/ankle); 2 = moderate/severe involvement in a single area (as above) or mild involvement in 2-3 areas; 3 = severe involvement in two areas or moderate involvement in several areas; 4 = severe involvement in all areas. Statistical significance was determined using the non parametric Mann-Whitney U test. Differences were considered significant when p < 0.05. Hausing treatment, and killing of the animals were carried out in Genoa (Italy) at The National Cancer Research Institute's animal facility (authorization of the Italian Ministry of Health no.146/2009-A) and followed national legislative provisions (Italian low no.116 of January 1992). The project (no. 299) regarding the studies reported in this manuscript was approved by the Ethical Committee for studies on animals and by the Italian Ministry of Health.

## List of abbreviations

UG: uteroglobin; TNF: Tumour Necrosis Factor; TNFRII: Tumour Necrosis Factor type II Receptor; FN: fibronectin; ED-B: extra domain B of fibronectin; RA: Rheumatoid Arthritis; ECM: Extra Cellular Matrix.

## Authors' contributions

EV has made substantial contributions to conception and design, acquisition, analysis and interpretation of data. EB, LB, BC, PC, GT have been involved in drafting the manuscript and revising it critically for important intellectual content. ZL has made substantial contributions to conception and design, has been involved in drafting the manuscript and has given the final approval of the version to be published. All authors read and approved the final manuscript.
